# Oral *Candida albicans *isolates from HIV-positive individuals have similar in vitro biofilm-forming ability and pathogenicity as invasive *Candida *isolates

**DOI:** 10.1186/1471-2180-11-247

**Published:** 2011-11-04

**Authors:** Juliana C Junqueira, Beth B Fuchs, Maged Muhammed, Jeffrey J Coleman, Jamal MAH Suleiman, Simone FG Vilela, Anna CBP Costa, Vanessa MC Rasteiro, Antonio OC Jorge, Eleftherios Mylonakis

**Affiliations:** 1 Department of Biosciences and Oral Diagnosis, Univ Estadual Paulista/UNESP, 777 Av. Eng. Francisco José Longo, São José dos Campos, SP 12245000, Brazil; 2 Division of Infectious Diseases, Massachusetts General Hospital, 55 Fruit Street, Boston, MA 02114, USA; 3 Emílio Ribas Institute of Infectious Diseases, 165 Av. Dr. Arnaldo, São Paulo, SP 01246900, Brazil

## Abstract

**Background:**

*Candida *can cause mucocutaneous and/or systemic infections in hospitalized and immunosuppressed patients. Most individuals are colonized by *Candida *spp. as part of the oral flora and the intestinal tract. We compared oral and systemic isolates for the capacity to form biofilm in an in vitro biofilm model and pathogenicity in the *Galleria mellonella *infection model. The oral *Candida *strains were isolated from the HIV patients and included species of *C. albicans*, *C. glabrata*, *C. tropicalis*, *C. parapsilosis*, *C. krusei*, *C. norvegensis*, and *C. dubliniensis*. The systemic strains were isolated from patients with invasive candidiasis and included species of *C. albicans*, *C. glabrata*, *C. tropicalis, C. parapsilosis, C. lusitaniae*, and *C. kefyr*. For each of the acquired strains, biofilm formation was evaluated on standardized samples of silicone pads and acrylic resin. We assessed the pathogenicity of the strains by infecting *G. mellonella *animals with *Candida *strains and observing survival.

**Results:**

The biofilm formation and pathogenicity in *Galleria *was similar between oral and systemic isolates. The quantity of biofilm formed and the virulence in *G. mellonella *were different for each of the species studied. On silicone pads, *C. albicans *and *C. dubliniensis *produced more biofilm (1.12 to 6.61 mg) than the other species (0.25 to 3.66 mg). However, all *Candida *species produced a similar biofilm on acrylic resin, material used in dental prostheses. *C. albicans*, *C. dubliniensis*, *C. tropicalis*, and *C. parapsilosis *were the most virulent species in *G. mellonella *with 100% of mortality, followed by *C. lusitaniae *(87%), *C. novergensis *(37%), *C. krusei *(25%), *C. glabrata *(20%), and *C. kefyr *(12%).

**Conclusions:**

We found that on silicone pads as well as in the *Galleria *model, biofilm formation and virulence depends on the *Candida *species. Importantly, for *C. albicans *the pathogenicity of oral *Candida *isolates was similar to systemic *Candida *isolates, suggesting that *Candida *isolates have similar biofilm-forming ability and virulence regardless of the infection site from which it was isolated.

## Background

Fungi are increasingly recognized as major pathogens in critically ill patients. *Candida *spp. are the fourth leading cause of bloodstream infections in the U.S. and disseminated candidiasis is associated with a mortality in excess of 25% [1-3]. Oropharyngeal candidiasis (OPC) is the most frequent opportunistic infection encountered in human immunodeficiency virus (HIV) infected individuals with 90% at some point experiencing OPC during the course of HIV disease [4]. Among *Candida *species, *C. albicans *is the most commonly isolated and responsible for the majority of superficial and systemic infections. However, many non-*albicans *species, such as *C. glabrata*, *C. parapsilosis *and *C. tropicalis *have recently emerged as important pathogens in suitably debilitated individuals [5].

A major virulence factor of *Candida *is its ability to adapt to a variety of different habitats and the consequent formation of surface-attached microbial communities known as biofilms [5]. *Candida *biofilms can develop on natural host surfaces or on biomaterials used in medical devices such as silicone and in dental prosthesis such as acrylic resin [6,7]. The biofilm formation in vitro entails three basic stages: (i) attachment and colonization of yeast cells to a surface, (ii) growth and proliferation of yeast cells to allow formation of a basal layer of anchoring cells, and (iii) growth of pseudohyphae and extensive hyphae concomitant with the production of extracellular matrix material [8,9]. Once established, *Candida *biofilms serve as a persistent reservoir of infection and are more resistant to antifungal agents [6].

The versatility of fungal pathogenicity mechanisms and their development of resistance to antifungal drugs indicate the importance of understanding the nature of host-pathogen interactions. Researchers have developed invertebrate model hosts in order to facilitate the study of evolutionarily preserved elements of fungal virulence and host immunity [10]. These invertebrate systems such as *Caenorhabditis elegans*, *Drosophila melanogaster, Dictyostelium discoideum *and *Galleria mellonella *offer a number of advantages over mammalian vertebrate models, predominantly because they allow the study of strains without the ethical considerations associated with mammalian studies [11-13]. Importantly, *Candida *pathogenicity can be evaluated using the greater wax moth *G. mellonella *as an infection model. This model has yielded results that are comparable to those obtained using mammalian models and there is remarkable commonality between virulence factors required for disease in mice and for killing of *G. mellonella *[14-17].

The pathogenesis of *Candida *spp. depends upon the coordinated expression of multiple genes in a manner that facilitates proliferation, invasion and tissue damage in a host. Since each invaded tissue is a unique ecological niche that changes over the course of the disease process, the expression of genes by *Candida *can vary according the infected site [18]. Costa et al. [19] demonstrated that blood *Candida *isolates were more proteolytic than oral cavity isolates while oral cavity isolates produced more phospholipase than blood isolates. On the other hand, Hasan et al. [20] using colorimetric assays verified that *C. albicans *strains isolated both from blood and oral mucosa produced the same quantity of biofilm. However, there are no studies to interrogate biofilm production on medical biomaterials and pathogenicity of isolates from localized and systemic candidiasis using an invertebrate model.

The objective of this study was to compare biofilm production of oral and systemic *Candida *isolates using an in vitro biofilm model on silicone (a material that is used in a number of implantable devices and catheters) and acrylic resin (a material that is used in preparation of dental prostheses). We were also interested in determining the pathogenicity of the strains in the *Galleria mellonella *infection model, considering they were isolated from different host environments, either blood or oral collection sites.

## Methods

### *Candida *isolates

A total of 33 clinical *Candida *strains recovered from oral and systemic candidiasis of different patients were used in this study. The oral *Candida *strains were isolated from the saliva or oropharyngeal candidiasis of 17 HIV-positive patients (65% men, 35% women) at the Emílio Ribas Institute of Infectious Diseases (São Paulo, SP, Brazil). The mean age was 46 years (33 - 63 years) and CD4+ lymphocyte counts ranged from 105 to 1000 cells/mm^3 ^with a mean count of 388 cells/mm^3^. *Candida *spp. isolated from these patients included: *C. albicans *(n = 11), *C. glabrata *(n = 3), *C. tropicalis *(n = 2), *C. parapsilosis *(n = 1), *C. krusei *(n = 1), *C. norvegensis *(n = 1), and *C. dubliniensis *(n = 2). The Ethics Committee of the Emílio Ribas Institute of Infectious Diseases approved this study (275/2009).

The systemic *Candida *strains were isolated from patients with invasive candidiasis at Massachusetts General Hospital (Boston, MA, USA) and included species of *C. albicans *(n = 5), *C. glabrata *(n = 2), *C. tropicalis *(n = 2)*, C. parapsilosis *(n = 1)*, C. kefyr *(n = 1), *and C. lusitaniae *(n = 1) (Table 1). These isolates were collected from eleven patients with a mean age of 57 years (40-78), that were HIV negative but had other underlying medical conditions. The use of *Candida *isolates was approved by the Massachusetts General Hospital Institutional Review Board (2008-P-001017).

**Table 1:** *Candida *isolates used in this study and their susceptibility to antifungals and interactions with *G. mellonella*

Microorganisms			Susceptibility to Antifungal (MIC)		*Galleria mellonella*		
**Specie of** ***Candida***	**Strain of** ***Candida***	**Clinical** **isolate**	**Fluconazole** **(μg/mL)**	**Amph B** **(μg/mL)**	**CFU/larva** **injected**	**Number of killing/total**	**Medium time to mortality (h)**

*C. albicans*	4S	Saliva	0.125	0.25	7.1 × 10^5^	16/16	18
	10S	Saliva	0.125	0.5	5.2 × 10^5^	16/16	18
	24S	Saliva	0.125	0.5	9.4 × 10^5^	16/16	18
	31S	Saliva	0.125	0.5	5.0 × 10^5^	16/16	24
	39S	Saliva	Resistant	0.25	5.9 × 10^5^	16/16	18
	48S	Saliva	0.125	0.25	6.7 × 10^5^	16/16	18
	60S	Saliva	0.125	0.25	6.3 × 10^5^	16/16	18
	3	OPC	0.5	0.25	7.2 × 10^5^	16/16	18
	14	OPC	Resistant	0.25	5.7 × 10^5^	16/16	18
	21	OPC	Resistant	0.25	7.5 × 10^5^	16/16	18
	37	OPC	Resistant	0.25	5.5 × 10^5^	16/16	18
	CAL006	Blood culture	0.125	0.5	1.9 × 10^5^	16/16	24
	CAL007	Peritoneal fluid	2	0.25	4.5 × 10^5^	16/16	24
	CAL008	Peritoneal fluid	1	0.5	7.2 × 10^5^	16/16	18
	CAL009	Blood culture	1	0.5	4.7 × 10^5^	16/16	18
	CAL010	Subdiaphragnatic	1	0.5	4.8 × 10^5^	16/16	18
*C. tropicalis*	12	OPC	0.5	0.25	3.9 × 10^5^	16/16	18
	140S	Saliva	0.125	0.25	4.9 × 10^5^	16/16	18
	CTR002	Synovial fluid	Resistant	0.5	9.1 × 10^5^	16/16	18
	CTR003	Abdominal fluid	2	0.5	5.0 × 10^5^	16/16	18
*C. parapsilosis*	127S	Saliva	1	0.5	6.2 × 10^5^	16/16	18
	CPA001	Lung tissue	4	0.5	7.3 × 10^5^	16/16	21
*C. glabrata*	12S	Saliva	2	0.5	6.4 × 10^5^	2/16	-
	45	OPC	4	0.5	9.8 × 10^5^	6/16	-
	55	OPC	4	0.5	1.0 × 10^6^	0/16	-
	CGL002	Drainage	32	0.5	4.0 × 10^5^	0/16	-
	CGL003	Jackson-Pratt fluid	32	0.5	5.4 × 10^5^	8/16	-
*C. dubliniensis*	18S	Saliva	16	0.25	3.9 × 10^5^	16/16	18
	155S	Saliva	0.5	0.25	5.1 × 10^5^	16/16	18
*C. lusitaniae*	CLU005	Blood culture	2	1	1.4 × 10^5^	14/16	-
*C.norvegensis*	52S	Saliva	32	0.5	6.3 × 10^5^	6/16	-
*C. krusei*	58	OPC	Resistant	2	8.8 × 10^5^	4/16	-
*C. kefyr*	CKE002	Blood culture	2	1	4.2 × 10^5^	2/16	-

The identification of *Candida *species was done by growth on Hicrome *Candida *(Himedia, Munbai, India), germ tube test, clamydospore formation on corn meal agar, and API20C for sugar assimilation (BioMerieux, Marcy Etoile, France). The identity of *C. dubliniensis *was determined by a multiplex polymerase chain reaction (PCR) procedure, according to the methodology described by MähB et al. [21].

Susceptibility patterns of the isolates to fluconazole and amphotericin B were determined by the broth microdilution assay according to the Clinical and Laboratory Standards Institute (CLSI) document M27-A2 [22]. Final concentrations of fluconazole ranged from 64 to 0.125 μg/mL and amphotericin B from 16 to 0.031 μg/mL. Antifungal activity was expressed as the minimum inhibitory concentration (MIC) of each isolate to the drug. The resistance breakpoints were used as described in the CLSI guidelines [22].

### In vitro biofilm model

The ability of *Candida *isolates to form biofilm on silicone and acrylic resin was evaluated as described by Nobile & Mitchell [23] and Breger et al. [24]. In brief, strains of *Candida *were grown in YPD medium (2% dextrose, 2% Bacto Peptone, 1% yeast extract) overnight at 30°C, diluted to an OD_600 _of 0.5 in 2 mL Spider medium, and added to a well of a sterile 12-well plate containing a silicone square measuring 1.5. × 1.5 cm (cut from Cardiovascular Instrument silicone sheets) or a chemically activated acrylic resin measuring 5 mm in diameter and 2.5 mm in thickness (Clássico, São Paulo, SP, Brazil) that had been pretreated overnight with bovine serum (Sigma-Aldrich). The inoculated 12-well plate was incubated with gentle agitation (150 rpm) for 90 min at 37°C for adhesion to occur. The standardized samples were washed with 2 mL PBS, and incubation was continued for 60 h at 37°C at 150 rpm in 2 mL of fresh Spider medium.

The platform and attached biofilm were removed from the wells, dried overnight, and weighed the following day. The total biomass (mg) of each biofilm was calculated by subtracting the weight of the platform material prior to biofilm growth from the weight after the drying period and adjusting for the weight of a control pad exposed to no cells.

The average total biomass for each strain was calculated from four independent samples. Statistical significance among the *Candida *species was determined by the analyses of variance (ANOVA) and the Tukey test using the Minitab Program. For comparison between oral and systemic *Candida *isolates, the Student t test was used. A *p-*value of less than 0.05 was considered significant.

### *Galleria mellonella *infection model

*G. mellonella *were infected with *Candida *as previously described by Cotter et al. [25], Brennan et al. [26] and Fuchs et al. [27]. In brief, *G. mellonella *caterpillars in the final instar larval stage (Vanderhorst, Inc., St. Marys, Ohio) were stored in the dark and used within 7 days from the date of shipment. Sixteen randomly chosen caterpillars (330 ± 25 mg) were infected for each *Candida *isolate.

*Candida *inocula were prepared by growing 50 mL YPD cultures overnight at 30°C. Cells were pelleted at 1,308 Xg for 10 min followed by three washes in PBS. Cell densities were determined by hemacytometer count. *Candida *inocula were confirmed by determining the colony-forming units per milliliter (CFU/mL) on YPD.

A Hamilton syringe was used to deliver *Candida *inocula at 10^5 ^cells/larvae in a 10 μL volume into the hemocoel of each larva via the last left proleg. Before injection, the area was cleaned using an alcohol swab. After injection, larvae were incubated in plastic containers (37°C), and the number of dead *G. mellonella *was scored daily. Larvae were considered dead when they displayed no movement in response to touch. Killing curves were plotted and statistical analysis was performed by the Log-rank (Mantel-Cox) test using Graph Pad Prism statistical software.

## Results

### Antifungal susceptibility of oral and systemic *Candida *isolates

The data of *Candida *strains identification and susceptibility to antifungal drugs (MIC) are shown in Table 1. The range of MIC to fluconazole was 0.125 to 64 μg/mL both for oral and systemic isolates. The resistance to fluconazole was observed in 5 (23%) oral isolates (4 *C. albicans *and 1 *C. krusei*) and 1 (8%) systemic isolate of *C. tropicalis*. The MIC to amphotericin B ranged from 0.25 to 2 μg/mL for oral isolates and from 0.25 to 1 μg/mL for systemic isolates.

### Biofilm formation by oral and systemic *Candida *isolates

**Figure 1:**
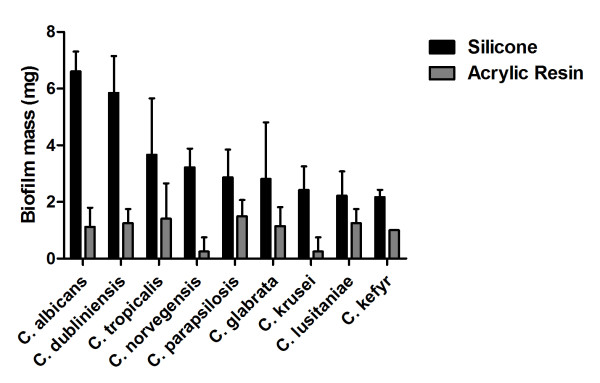
**Means and SDs of the biofilm mass formed on silicone pads and acrylic resin for *Candida *species studied**.

All isolates of oral and systemic candidiasis formed biofilm on silicone pads, but the quantity of biofilm mass was different for the species studied ranging from 2.17 to 6.61 mg. Biofilm formation was highest in *C. albicans *and *C. dubliniensis *followed by *C. tropicalis *and *C. norvegensis*. Biofilm mass formed by *C. albicans *differed significantly from biofilm mass produced by *C. norvegensis *(*P *= 0.009), *C. parapsilosis *(*P *= 0.003), *C. glabrata *(*P *= 0.001), *C. krusei *(*P *= 0.001), *C. lusitaniae *(*P *= 0.001), and *C. kefyr *(*P *= 0.001). Biofilm produced by *C. dubliniensis *was significantly different from biofilm mass produced by *C. parapsilosis *(*P *= 0.046), *C. glabrata *(*P *= 0.025), *C. krusei *(*P *= 0.013), *C. lusitaniae *(*P *= 0.007), and *C. kefyr *(*P *= 0.006) (Table 2 and Figure 1).

**Table 2:** Means and SDs of the biofilm mass (mg) formed on silicone pads and acrylic resin for *Candida *species studied and p-value obtained for each *Candida *specie compared to *C. albicans *(Tukey test, *P *< 0.05)

*Candida *species	Silicone	p-value (compared to *C. albicans*)	Acrylic resin	p-value (compared to *C. albicans*)
*C. albicans*	6.61 ± 0.70	-	1.12 ± 0.68	-
*C. tropicalis*	3.66 ± 2.22	0.062	1.41 ± 1.25	0.998
*C. parapsilosis*	2.87 ± 0.98	0.003	1.50 ± 0.57	0.982
*C. glabrata*	2.81 ± 2.09	0.001	1.15 ± 0.67	1.000
*C. dubliniensis*	5.85 ± 1.30	0.989	1.25 ± 0.50	1.000
*C. lusitaniae*	2.22 ± 0.86	0.001	1.25 ± 0.50	1.000
*C. norvegensis*	3.22 ± 0.66	0.001	0.25 ± 0.50	0.347
*C. krusei*	2.42 ± 0.84	0.001	0.25 ± 0.50	0.347
*C. kefyr*	2.17 ± 0.26	0.001	1.00 ± 0.00	1.000

The biofilm mass adhered on acrylic resin ranged from 0.25 to 1.50 mg depending on the *Candida *species tested. *C. albicans*, *C. dubliniensis*, *C. tropicalis*, *C. parapsilosis*, *C. glabrata*, and *C. lusitaniae *formed more biofilm than *C. norvegensis*, *C. krusei *and *C. kefyr*. However, significant differences between the *Candida *species were not observed (*P *= 0.062) (Table 2 and Figure 1).

**Figure 2:**
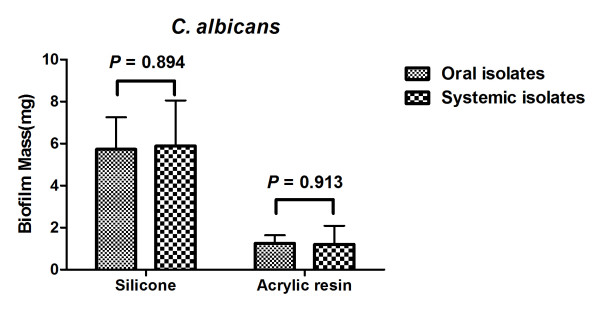
**Means and SDs of the biofilm mass formed on silicone pads and acrylic resin for oral and systemic *Candida *isolates**. Statistical analysis was performed using a Student t-test.

The biofilm mass formed by oral and systemic isolates of *C. albicans *were compared and showed similar results both for biofilm formed on silicone pads as biofilm formed on acrylic resin (Figure 2).

### Killing of *G. mellonella *by oral and systemic *Candida *isolates

**Figure 3:**
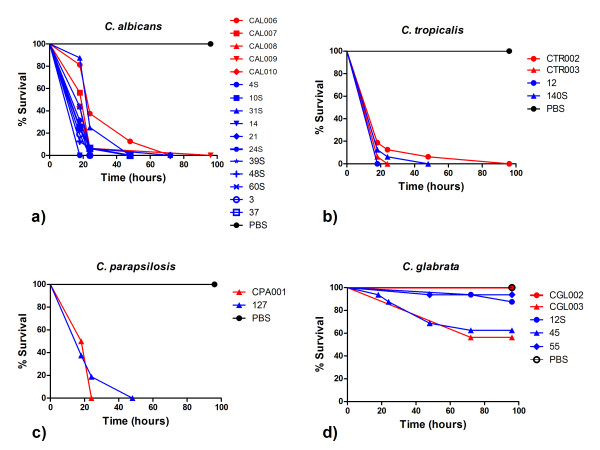
**Killing of *G. mellonella *larvae by oral (blue lines) and systemic (red lines) isolates of *Candida***. Comparison of killing curves by Log-rank test: a) strains of *C. albicans *(*P *= 0.372); b) strains of *C. tropicalis *(*P *= 0.914); c) strains of *C. parapsilosis *(*P *= 0.661); d) strains of *C. glabrata *(*P *= 0.006). Injections with PBS were used as a control group.

**Figure 4:**
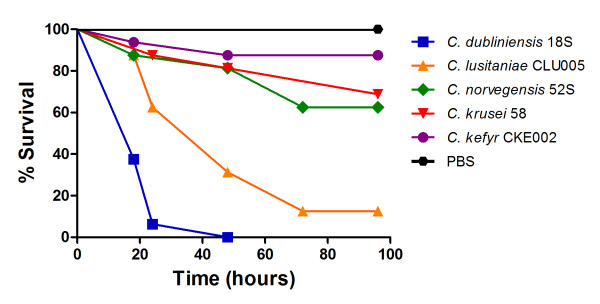
**Killing of *G. mellonella *larvae by isolates of *C. dubliniensis, C. lusitaniae, C. norvegensis, C. krusei*, and *C. kefyr***. Injections with PBS were used as a control group.

The virulence of *Candida *isolates in the *G. mellonella *model was dependent on the species studied. *C. albicans*, *C. dubliniensis*, *C. tropicalis and C. parapsilosis *were the most virulent species in *G. mellonella *(Table 1). Among all *Candida *strains studied, *G. mellonella *showed mortality rates of 100% after injection with *C. albicans*, *C. dubliniensis*, *C. tropicalis*, and *C. parapsilosis*, 87% with *C. lusitaniae*, 37% with *C. novergensis*, 25% with *C. krusei*, 20% with *C. glabrata*, and 12% with *C. kefyr *over a 96 hour period (Figures 3 and 4). Of note is that, all isolates of *C. albicans*, including strains sensitive and resistant to fluconazole, presented the same virulence in *G. mellonella *with a medium time to mortality of 18 to 24 hours (Table 1).

The virulence between oral and systemic *Candida *isolates was compared according to each species of *Candida*. The results of survival of *G. mellonella *larvae showed no statistically significant difference between oral and systemic isolates of *C. albicans *(*P *= 0.372, Figure 3a), *C. tropicalis *(*P *= 0.914, Figure 3b), and *C. parapsilosis *(*P *= 0.661, Figure 3c).

For *C. glabrata*, a statistically significant difference was observed between the strains CGL002 and CGL003 (*P *= 0.003), CGL002 and 45 (*P *= 0.007), CGL003 and 12S (*P *= 0.049), CGL003 and 55 (*P *= 0.024), 45 and 55 (*P *= 0.033), showing the occurrence of variation in virulence between strains of *C. glabrata *for both the oral isolates and the systemic isolates (Figure 3d).

## Discussion

In this study we compared the pathogenicity of oral and systemic *Candida *isolates. The pathogenesis of diverse candidal diseases depends upon both generalized virulence factors and those that function in specific environments dictated by immune function, tissue site and other host factors. The data presented here demonstrate that the pathogenicity of oral *Candida *isolates is similar to systemic *Candida *isolates, suggesting that the pathogenicity of *Candida *is not correlated with the infected site.

The pathogenesis of both oral and systemic candidiasis is closely dictated by properties of the yeast biofilms [28,29]. Implanted devices, such as venous catheters or dental prosthesis, are a serious risk factor for *Candida *infections. They are substrates for the formation of biofilm, which in turn serve as reservoirs of cells to continually seed an infection [8]. It has been estimated that at least 65% of all human infectious are related to microbial biofilms [30,31].

A variety of methods have recently been used for the quantification of *Candida *biofilm on different substrata. These include counting of colony forming units (CFU), dry-weight assays, spectrophotometric analysis, and colorimetric assays, such as 2,3-bis (2-methoxy-4-nitro-5-sulfophenyl)-5-[(phenylamino) carbonyl]-2H-tetrazolium hydroxide (XTT) reduction assay. However, each method carries its own advantages and limitations [7,32,33]. In our study, we used a dry-weight assay because this method allows the single quantification of a *Candida *biofilm on a clinically relevant substrate such as silicone and acrylic resin. Silicone is frequently used in the manufacture of medical devices and catheters and it is related to development of systemic candidiasis in hospitalized patients. Acrylic resin (methyl methacrylate) is a material widely used in preparation of dental prosthesis and it has significance for development of oral candidiasis.

Among all isolates tested in this study, the quantity of biofilm mass varied according to the *Candida *species. *C. albicans *and *C. dubliniensis *were the highest biofilm producers on silicone pads, followed by *C. tropicalis*, *C. norvegensis, C. parapsilosis, C. glabrata, C. krusei, C. lusitaniae*, and *C. kefyr*. Most studies have shown that the biofilm formation by clinical isolates of *Candida *was species dependent and generally the highest levels of biofilm formation were observed in *C. albicans *and the lowest in *C. glabrata *[5,20]. Notably, unlike *C. albicans *and other *Candida *species, *C. glabrata *is unable to generate filamentous forms which may contribute to the impared ability of *C. glabrata *to form a biofilm [5]. The observations for higher quantities of biofilm production by *C. albicans *and lower biofilm production from the non filamenting *C. glabrata*, given the same standards of in vitro test conditions, remained true for the clinical isolates from our study. Indeed, for both strains collected orally or systemically, there was very little in the way of quantity or quality of biofilm production for *C. glabrata*. *C. albicans *produced the greatest quantity of biofilm regardless of the adhesion platform material or whether it was isolated from oral or invasive infection sites.

Interestingly, the differences in biofilm formation among *Candida *species on acrylic resin were less significant than biofilm formed on silicone. This fact may be attributed to the methodology used which was previously developed for biofilm formation on silicone pads [23,24]. The process of candidal adhesion to acylic resins is complex. Previous studies have shown that a number of factors including the nutrient source, the sugar used for growth (glucose or sucrose), and the formation of pellicules from saliva or serum may influence the adhesion and colonization of *Candida *[7,29].

We also used an in vivo *G. mellonella *infection model to evaluate the pathogenicity of oral and systemic *Candida *isolates. There are some benefits to using *G. mellonella *larvae as a model host to study *Candida *compare to other invertebrate models. For example, the larvae can be maintained at a temperature range from 25°C to 37°C, thus facilitating a number of temperature conditions under which fungi exist in either natural environmental niches or mammalian hosts. High temperatures can be prohibitive for the growth of *C. elegans *or *Drosophila infection *models. Our study used 37°C to mimic mammalian infection systems. *G. mellonella *also has the benefit of facile inoculation methods either by injection or topical application, where injection inoculation provides a means to deliver a precise amount of fungal cells [12,27,34]. By contrast, other systems, such as *C. elegans*, require infection through ingesting the pathogen. Since we included both *albicans *and non-*albicans *strains in our study we thought it prudent to use a model that ensured equal pathogen delivery rather than a model that would have an aversion to consuming some of the infecting agents.

As with the biofilm assays, the virulence levels of *Candida *isolates in *G. mellonella *were dependent on the species studied. Surprisingly, within the same species, oral isolates were as virulent as isolates from candidemia, the most common severe *Candida *infection. Previously, Cotter et al. [25] reported that it is possible to distinguish between different levels of pathogenicity within the genus *Candida *using *G. mellonella *larvae. We observed that *G. mellonella *showed mortality rates of 100% after injection with 10^5 ^cells of *C. albicans*, *C. dubliniensis*, *C. tropicalis*, and *C. parapsilosis*, 87% with *C. lusitaniae*, 37% with *C. novergensis*, 25% with *C. krusei*, 20% with *C. glabrata*, and 12% with *C. kefyr *over a 96 hour period of incubation at 37°C. Cotter et al. [25] verified mortality rates of 90% for *C. albicans*, 70% for *C. tropicalis*, 45% for *C. parapsilosis*, 20% for *C. krusei*, and 0% for *C. glabrata *over a 72 hour period of incubation at 30°C after the injection with 10^6 ^cells of each *Candida *species. Probably, the virulence of the *Candida *strains in *G. mellonella *tested in this study were higher than the virulence of *Candida *strains observed by Cotter et al. [25] because of the difference of incubation temperature used. The temperature variations can affect gene expression and consequently the level of virulence of *Candida *strains [35].

Of note is that this is the first study to inoculate species of *C. lusitaniae*, *C. norvegensis *and *C. dubliniensis *in the *G. mellonella *model. Single isolates for *C. lustaniae *and *C. norvegensis *and two isolates of *C. dubliniensis *were included in our study. *C. lusitaniae *is considered an emerging non-*albicans Candida *species and isolates show resistance to amphotericin B. *C. norvegensis *appears to be a rare cause of human infection and the most of the isolates are resistant to fluconazole [36,37]. There are limited data on the comparative virulence of *C. lusitaniae *and *C. norvegensis *in relation to *C. albicans*. In this study, *C. lusitaniae *and *C. norvegensis *were less virulent in *G. mellonella *than *C. albicans*.

Finally, in our study, *C. dubliniensis *isolates showed that the ability of biofilm formation and killing *G. mellonella *was similar to *C. albicans. C. dubliniensis *has been implicated in oropharyngeal candidiasis in HIV-infected patients, althought it has also been isolated from other anatomical sites, including lungs, vagina, blood, and feces [38,39]. Despite the significant phenotypic and genotypic similarities shared between *C. albicans *and *C. dubliniensis*, the comparative virulence of the two species is clearly a very complex topic [40,41]. Borecká-Melkusová [42] verified that the biofilm formation in *C. albicans *was significantly lower than in *C. dubliniensis*, and Koga-Ito et al. [43] observed that the survival rate and dissemination capacity of *C. dubliniensis *in mice were lower than *C. albicans*.

## Conclusion

In summary, in *Candida *spp., the ability of biofilm formation and virulence in the *G. mellonella *model were dependent on the species studied. For *C. albicans *the pathogenicity of oral isolates was similar to that of systemic isolates, suggesting that oral *Candida *infections should be taken seriously as they have the potential to be as equally morbid if they become systemic infections. Of note is that the penetration by *C. albicans *filaments is critical during the course of the infection in the *Galleria *tissue [17]. However, this model does not focus on invasion. Further studies are needed in order to study the ability of oral isolates to colonize and penetrate tissues.

## Authors' contributions

JCJ and EM participated in the design, implementation, analysis, interpretation of the results and wrote this manuscript. JMAHS collected the *Candida *strains from the oral cavity of HIV-positive patients. SFGV, ACBPC, VMCR and AOCJ performed the identification and the antifungal susceptibility of oral *Candida *isolates. BBF participated in the in vitro biofilm model and helped to draft the manuscript. MM participated in the *G. mellonella *assays. JJC identified the systemic *Candida *isolates. All authors read and approved the final manuscript. The authors declare no conflict of interest.
